# The Role of Community and Social Support in the Link Between Social Determinants of Health and Self-Rated Health

**DOI:** 10.1093/geroni/igaf122.2020

**Published:** 2025-12-31

**Authors:** Samuel Van Vleet, Jeein Law (Jang, John Bowblis

**Affiliations:** Miami University, Oxford, Ohio, United States; University of Massachusetts Boston, Boston, Massachusetts, United States; Miami University, Oxford, Ohio, United States

## Abstract

As the older adult population in the United States grows, understanding how social determinants of health, neighborhood environment, and social networks influence health outcomes in later life is crucial. Marginalized populations often face cumulative disadvantages, making community support essential for improving health. This study explores the impact of social determinants of health, neighborhood environment, and social networks on self-reported health among older adults with multiple chronic conditions, using data from the National Health and Aging Trends Study (N = 5,356). Survey-weighted logistic regression models assessed relationships between income, rurality, food insecurity, transportation, meal assistance, neighborhood disorder, and built environment issues, with self-rated health as the outcome. The moderating effects of community support and social network size were also examined. Results indicate that food insecurity, transportation difficulties, limited meal assistance, and high physical disorder were associated with lower self-rated health, while higher community support was associated with better self-rated health. Notably, community support buffered the negative impact of food insecurity and high physical disorder on self-rated health. Additionally, social network size positively moderated the negative impact of high physical disorder on self-rated health. These findings emphasize the significant role of social determinants of health and social support in shaping health outcomes in later life. Policies should prioritize strengthening community support systems and social networks, particularly for food-insecure and physically disadvantaged older adults, to improve self-rated health and mitigate health disparities. The study contributes to understanding how social and environmental factors intersect to affect health disparities among aging populations.

